# Promoting Health in Virtual Worlds: Lessons From Second Life

**DOI:** 10.2196/jmir.3177

**Published:** 2014-10-13

**Authors:** Reima Suomi, Matti Mäntymäki, Sari Söderlund

**Affiliations:** ^1^Institute of Information Systems ScienceDepartment of Management and EntrepreneurshipTurku School of EconomicsTurkuFinland; ^2^Finland Futures Research CenterTurku School of EconomicsUniversity of TurkuTurkuFinland

**Keywords:** social media, Second Life, virtual worlds, health, health promotion

## Abstract

**Background:**

Social media services can help empower people to take greater responsibility for their health. For example, virtual worlds are media-rich environments that have many technically advantageous characteristics that can be used for Health 2.0 purposes. Second Life has been used to build environments where people can obtain information and interact with other users for peer support and advice from health care professionals.

**Objective:**

The intent of the study was to find out whether Second Life is a working and functional platform supporting the empowerment of people in health-related issues.

**Methods:**

We conducted a review of the current health-related activity in Second Life, coupled with an extensive series of observations and interactions with the respective resources inside Second Life.

**Results:**

A total of 24 operative health resources were found in Second Life, indicating that health-related activity is rather limited in Second Life, though at first glance it appears to contain very rich health-related content. The other main shortcomings of Second Life relate to a lack of activity, a low number of resource users, problems with Second Life’s search features, and the difficulty of finding trustworthy information.

**Conclusions:**

For the average user, Second Life offers very little unique value compared to other online health resources.

## Introduction

### Importance of the Topic

Health 2.0 and Medicine 2.0 have become topical concepts in fields such as medicine, behavioral science, nursing studies, and information systems, to name a few [1-3]. Health 2.0 refers to “the use of a specific set of Web tools (blogs, podcasts, tagging, search, wikis, etc) by actors in health care, including doctors, patients, and scientists, using principles of open source and the generation of content by users, and the power of networks in order to personalize health care, collaborate, and promote health education”[3]. These concepts address the role of the individual in taking greater responsibility for their health by utilizing technologies such as social media [4], defined as “a group of Internet-based applications that build on the ideological and technological foundations of Web 2.0, which allows the creation and exchange of user-generated content” [5].

For example, virtual worlds, defined as persistent computer-mediated 3-D environments where the users are represented as avatars [6], are a subset of social media [5] that have been used to build environments in which people can obtain and exchange health-related information. By offering a wide range of audio-visual stimuli in the media-rich, multi-user environment, they are able to create an immersive user experience and simulations of reality.

The characteristics of virtual worlds—the possibility to present oneself anonymously as an avatar, experience things, and interact with others in a virtual environment—can empower people with motoric or sensory disabilities and chronic illnesses that can hamper movement in the physical environment and face-to-face social interactions. For example, the virtual world platform Second Life hosts a wide range of health-related activities from virtual operating theatres for the purpose of education to places where people can look for information and receive support. Consequently, virtual worlds can help to empower individuals to take greater responsibility for their personal health and well-being [7].

Interestingly, despite their advantageous technical characteristics, only a little research has explored virtual worlds from the empowerment perspective. In more general terms, research about social media in health care is still in its infancy and lags far behind practice [8], although Beard et al [9] did survey health-related activities in Second Life when there was considerable hype around virtual worlds and Second Life.

Against this backdrop, the aim of this paper is twofold: first to conduct a follow-up study to Beard et al [9] to gain updated insight on the health-related activity in Second Life at a time when the hype around virtual worlds has largely vanished. Second, we focus on investigating how Second Life has been used for patient/citizen empowerment purposes. To this end, we have conducted an extensive set of observations in Second Life.

The remainder of the paper is organized as follows: after the introduction, we discuss patient empowerment and prior applications of Second Life in health care. In the third section, we present our empirical research and the results. In the fourth section, we discuss the implications of the results, present the limitations of the study, and suggest areas for further inquiry.

### Background

#### Patient Empowerment With Social Media

People use social media in health care mainly for the purposes of sharing health care information and experiences with others, making effective decisions about health, and realizing the self-management of personal well-being. In the literature, the benefits of social media for the patient have been found to be: seeking emotional help or support, obtaining health information, exchanging information and experiences, seeking guidance online about health information and services, developing a different relationship with their doctors, health education and learning, and the self-management of health [1,2,10-14]. Social media has also been found to be helpful for different patient groups, such as teens with special health care needs as well as people with HIV, cancer, and chronic diabetes [13-15].

Social media can enable patients to become active and responsible partners in their health care activities [16]. As many diseases (eg, obesity, depression) are socially constructed, a key to their healing is also in social activity [17]. Thus, social media has been argued to have the potential to result in empowerment outcomes for patients wishing to improve communication with other patients and health professionals [18]. According to Eysenbach [4], patients using social media are empowered to contribute to and utilize the collective wisdom and experience of others to support their health care decisions and improve their health care.

#### Second Life and Health Care

Beard et al [9] surveyed health-related activities within Second Life in 2008. They discovered 68 health-related services in Second Life and concluded that the most common types of health-related resources in Second Life were those whose principle aim was to educate or offer information to patients or to increase awareness about health issues.

Yellowlees and Cook [19] investigated how Second Life can be used to increase awareness of severe mental illness. They constructed a visual environment to simulate the auditory and visual hallucinations of two patients with schizophrenia. A total of 863 survey respondents journeyed through the environment. Of those respondents, 440 (76%) thought the environment improved their understanding of auditory hallucinations, 69% thought it improved their understanding of visual hallucinations, and 82% said they would recommend the environment to a friend [19].

Boulos et al [1] evaluated the potential of 3-D environments in general in medical and health education and focused specifically on two resources in Second Life: Second Life Medical and Consumer Health Libraries and VNEC (Virtual Neurological Education Centre). They concluded (p. 242) that “3-D virtual worlds offer great potential to creative medical and health educators and librarians, but more research is needed into their use in medical and health education”.

**Figure 1:**
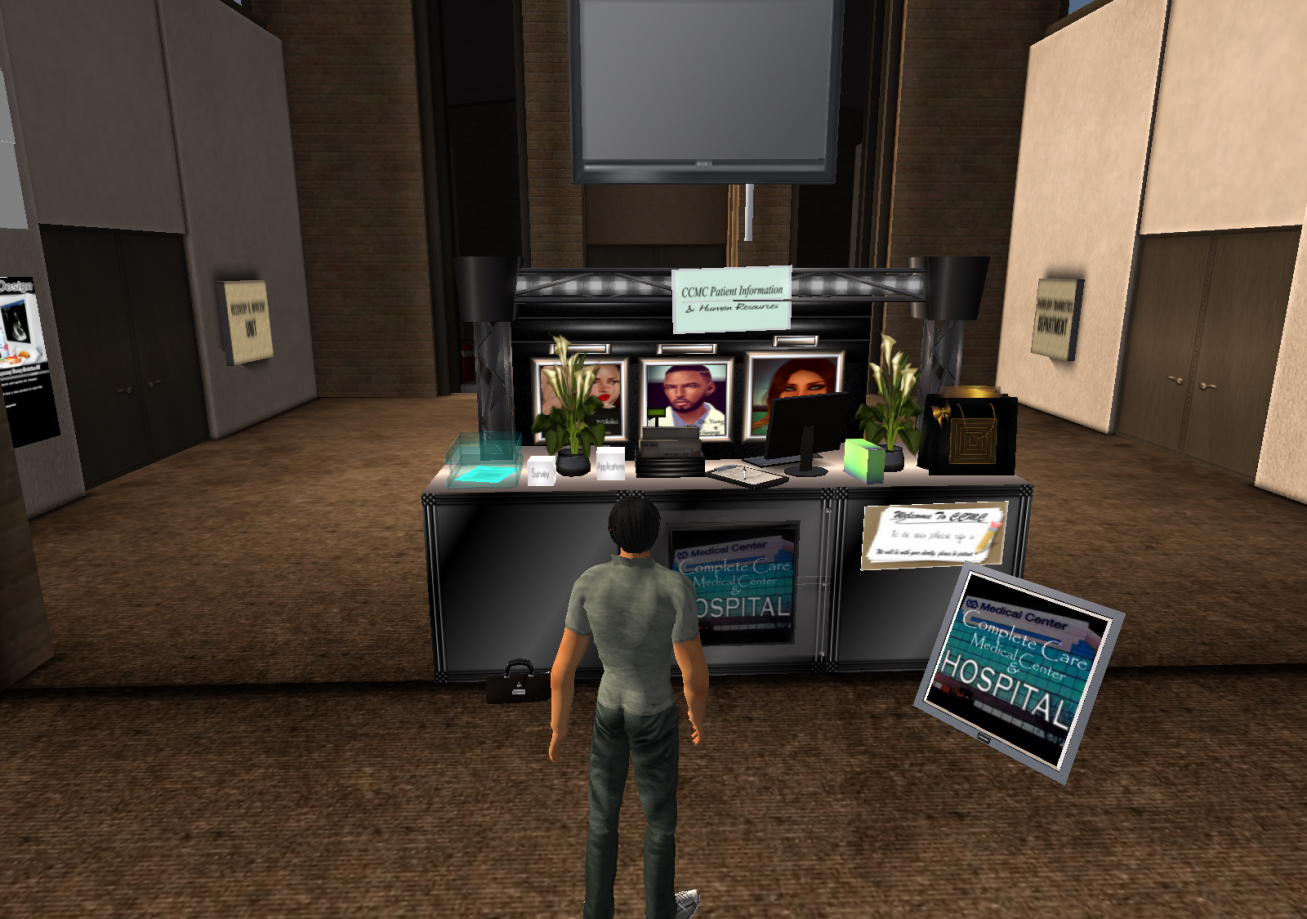
Reception desk of Complete Care Medical Center Hospital.

The literature has also examined how Second Life could be used in treating patients. Gorini, Gaggioli, Vigna, and Riva [20] discussed the use of Second Life for psychotherapy. They found that compared to conventional telehealth applications, such as email, chat, and videoconferencing, the interaction between real and 3-D virtual worlds may convey greater feelings of presence between the therapists. In addition, Second Life was able to better facilitate the clinical communication process, positively influence group processes and cohesiveness in group-based therapies, and foster higher levels of interpersonal trust between patients (p. 21). Figure 1 is a screenshot of the reception desk of Complete Care Medical Center Hospital.

## Methods

### Analysis of Current Health Activity in Second Life

#### Data Collection Phase

We collected the empirical data from health-related resources in Second Life. We first used the search features of Second Life as well as standard Internet searches to identify the health-related resources in Second Life. In the second stage of the empirical research, we conducted an extensive set of participant observation by accessing the resources we identified with our avatars. The data collection was initiated in late 2012 and concluded in June 2013.

We started our analysis by using the search features of Second Life (Second Life Destination Guide) with “health” as a search term. In addition, we did a similar search with other relevant keywords such as “hospital”, “medicine”, and “medical” to ensure we did not miss relevant results. Interestingly, we also found that the internal search features of Second Life returned slightly different numbers of results for analogous searches. Using the search features of Second Life, a total of 294 resources were found with the keyword “health”. Interestingly, in the Destination Guide of Second Life, using “health” as a search term did not return any results. In addition, we used standard Internet searches to obtain additional information about the resources we identified. This turned out to be very useful in evaluating the resources and further validating our findings.

We found that SL Healthy is a key resource within Second Life for health issues. The site itself describes itself as follows: “SL Healthy gathers information about consumer health locations and groups in Second Life, with general health education resources as well”. According to SL Healthy, there were 292 health resources in Second Life at the time of the data collection.

In addition, SL Science Center Group keeps track of information related to science and technology. SL Science Center Group list enumerates 11 resources with the title “clinic” and 2 resources with the title “health” (the list can be found with a Google search, not from Second Life). There is also a site nominating the top 10 Virtual Medical Sites in Second Life, although it was last updated in 2007.

To identify more relevant resources, we followed links, such as links to other Second Life locations, when applicable. For example, a good way to find new health-related activities was to follow other groups that the founders of health-related groups had belonged to. This approach is analogous to snowball sampling used in conducting a systematic literature review [21-23].

An overview of all health-related resources found in Second Life is presented in Multimedia Appendix 1.

#### Data Cleaning Phase

The list of results from the Second Life search included parcels and groups. Parcels represent areas of virtual land in Second Life. They have required a financial input from their owners and their traffic is measured. Groups can be put up free of charge, for example, around a topic of common interest and their activity level is impossible to follow without joining the groups.

The first cleaning task was to eliminate duplicates from the lists. This was difficult because the same resources sometimes appeared with slightly different names in different lists, and some resources formed nested structures.

**Figure 2:**
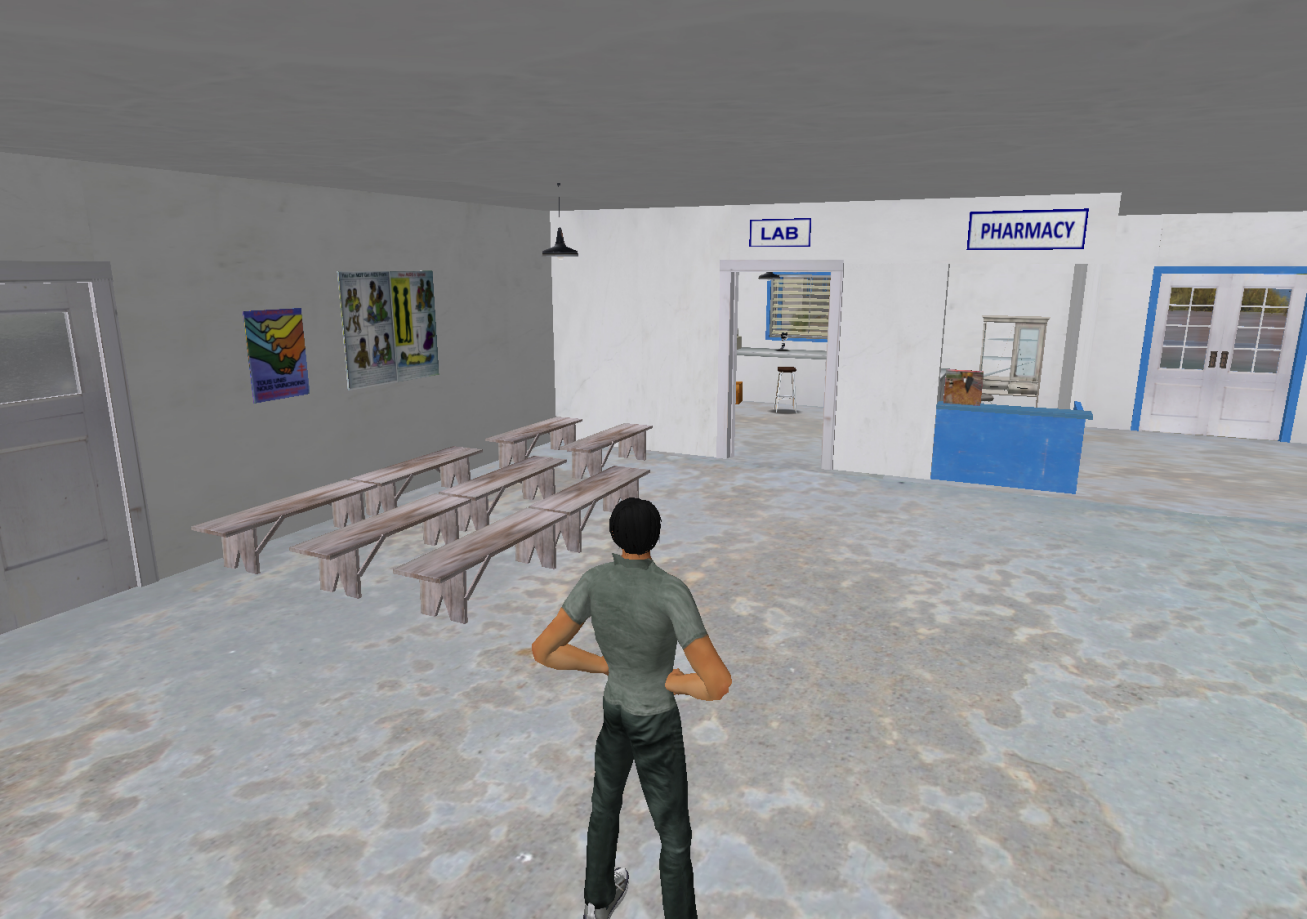
Entrance of Global Health.

After that, we excluded land parcels with a traffic rate of 20 or less based on Second Life statistics. This was due to the fact that these resources, although appearing in the search results, were however more or less “dead” with no customers. See Figure 2 for a screenshot of the entrance to Global Health.

After having established the final list of relevant resources, four researchers entered Second Life and independently accessed the listed resources and tabulated their findings. To systematize the analysis of the resources, we first adopted the classification of eHealth services by MacDonald, Case, and Mertzger [24] (see Multimedia Appendix 2). It soon became apparent that not all the components of the classification were applicable to our research context. For example, most of the groups were targeted to people who were both wealthy and ill, and material for prevention and cure was presented interchangeably. Thus, the researchers made notes about their observations and reflections when accessing the resources and saved material such as links and images in the project repository.

Thereafter, a final review and consolidation of the results was conducted. In the final stage, only 24 health-related active resources were identified and included for a further in-depth analysis as presented below.

## Results

Much of the activity in Second Life happens in different groups. Table 1 presents their typical themes. Typically these resources were designed to raise awareness and provide information on a specific topic, such as pregnancy, alcoholism, or Asperger’s syndrome. In addition, many resources offered some form of peer support.

Resources promoting sexual health appeared in the results. However, many of the sexual health-related resources were put up to promote the rights of people with different sexual orientations and create venues for social interaction between the members of these groups. Hence, deciding whether to include the theme of sexual health or not, and evaluating the degree of health orientation of this resource as well as others like it, was a challenging task.

Furthermore, there were also a handful of hospitals in Second Life. Their resources were often built to present new hospital concepts or to educate medical and nursing students on how to conduct medical procedures. Interestingly, it is rare for real life hospitals and private health companies to have a Second Life presence. This observation is in line with the findings by von Krogh, Jäger, and Barnetta [25] on the presence of Fortune 500 companies in Second Life.

A small group of resources concentrated on selling health-related items. These can be broadly divided into two very different categories: sellers of professional medical equipment such as instruments, clothing, and medical devices, and sellers offering different kinds of general products, such as scents, which they claimed contained therapeutic components. Interestingly, we did not observe any virtual pharmacy. We also observed resources for collecting money for charitable purposes, such as a resource offering residences, with the revenues being directed to medical research.

We also listed resources built around a specific illness or medical condition. Table 2 offers an overview of those resources.

We found that the most active resources were networked in several ways. For example, a single institution might have set up several brands or “entrance points” to their services in real life and on the Internet. Additionally, many of the resources seemed to alter their names quite often, which caused additional challenges in the analysis.

Universities seem to be the backbone of health and medical resources within Second Life because the platform is mainly used for teaching purposes. For example, the virtual hospital of Imperial College London offers a variety of simulated patient experiences for teaching students sequences of activities from the arrival of a patient.

One of the most interesting findings from our observations, when visiting the resources, was the lack of social interaction and the absence of other users. As Second Life is a very rich communication environment for facilitating the transmission of various social cues, such as facial expressions, gestures, and even voice, it offers many options for social interaction. From this perspective, it was surprising how little interaction we encountered during our observations. Most of the resources we visited were empty, except the welcoming robot greeting everyone entering the area. These observations do not mean that the resources are empty all the time; however, a lack of other users significantly reduces opportunities for peer support.

**Table 1:** Examples of health-related groups in Second Life.

Type of resource	Description
Maternity	Information about pregnancy and maternity
Alcohol	Peer support and information for people suffering from alcohol abuse
Disabilities	Information and peer support for people with disabilities
Donations	Collecting money to support health-related activities and organizations

**Table 2:** Examples of illnesses and medical issues present in Second Life.

Illness/medical issue	Description
Cancer	Information about cancer treatments for patients; peer support for their families
Autism	General information; several groups
Dementia	General information
Diabetes	General information; peer support; information for parents of children with Type 1 diabetes
Mental health	Information about various mental health problems, eg, bipolar disorder, anxiety, and depression as well as the simulation of schizophrenia
Physical disabilities	Information, particularly for people using wheelchairs

## Discussion

### Comparison With Prior Research

In their study, Beard et al [9] discovered 68 health-related services in Second Life. The years between 2007 and 2008 are considered to be peak years of Second Life activity, since then the amount of users of health-related Second Life media have declined. Our study found just 24 active resources, confirming this trend.

Today, health-related activity in Second Life concentrates on issues such as accessibility, mental health, societal health, and peer-to-peer support. This matches current developments and needs in mainstream medicine, such as the Citizen-Centric Care paradigm [26-28]. In this line of thinking, health is essentially co-produced by individuals and their communities.

### Key Findings

The main finding of this research was that virtual worlds such as Second Life can help people with illnesses and disabilities explore different things and interact with other people. In this sense, virtual worlds have the potential to help make societies more democratic by empowering people who have difficulties in real life. Furthermore, sensor-related disabilities can be compensated for by smart computer interfaces. Accordingly, a considerable part of the health-related activity in Second Life revolves around people with disabilities.

An interesting observation was that many of the health-related resources in Second Life were shut down or the level of activity appeared to be rather low. The technical capabilities of Second Life for offering users media-rich, immersive experiences in interaction with other users were not fully utilized. In most instances, we did not meet any other users. This indicates that either Second Life or the maintainers of the health-related resources inside the virtual world have not been very successful in promoting the sustained usage of their services. This is well in line with the findings of Miller and Tucker [29], who found that much of social media traffic is generated by the social media-initiating firms themselves, not by real customers. If we consider that sustained usage, also referred to as continuance, has been viewed as a critical success factor for online services [30], the challenge of attracting users who can sustain services poses a major threat to Second Life.

Second Life has existed for only 10 years. The track records of most health care resources in Second Life give a picture of a haphazard entrance to the platform—one that has little emphasis on building long-term relationships with users and promoting continuance. An example of the lack of strategic orientation is that many resources have changed their names, which is obviously not an ideal approach when building a brand and awareness of it. Our research confirms the findings of Korda and Itani [31] that social media seldom achieve their desired outcomes in the health care field. A related challenge is contacting customers who have avatars that do not necessarily have any connection to their real-life identities. Building long-term relationships generally requires that the parties exchange information about themselves to develop trust [32]. Social media should be giving patients opportunities to assess the quality of health care offerings [33], but contradictory Second Life sites seem not to care much about their quality.

Our results portray a picture of Second Life as a dying environment full of deserted places and “ghost towns”. In most health-related resources, most or even all activities date back to between 2006 and 2008—more recent entries were hard to find. It must be noted that keeping time stamps is not one of the strengths of Second Life, which leads to the observation: what is a society that does not maintain its history or a track of time?

### Limitations and Further Research

Our study has a number of limitations. First of all, as the environment constantly changes, our study is able to offer only a snapshot of a limited period of time. Second, determining the boundaries of the research area was problematic. For instance, does a discussion group on the beneficial effects of using horses to improve mental health fall within the research area? Much of the health-related activity in Second Life takes place in discussions between users, but these are not accessible without joining the (numerous) groups, so this method of validating the health-orientation of the resources was left unused in this research. In this study, we also focused only on the structures that have been built within the environment. Thus, a promising area for further research would be to conduct an ethnographic approach and observe active users, their communication patterns, and social structures in a longitudinal study. Third, in this study we focused solely on Second Life. However, there are a number of other virtual worlds that include health-related resources or facilitate health-related activities. For example, a leading social virtual world for teens, Habbo Hotel [34] has facilitated nurse receptions to offer its young users an opportunity to ask health-related questions from a trained professional. Hence, we suggest future research with broader contextual coverage.

### Conclusions

Based on the findings of this study, we argue that Second Life should be viewed as a stage in the evolution of virtual worlds. Since its inception, Second Life has educated large numbers of people about how to behave in the virtual environment and how to build a virtual presence. Today, its once innovative virtual world components, such as avatars and 3-D graphics, can be relatively easily implemented in any other online services.

Hence, we suggest that service developers analyze the shortcomings of Second Life in order to build better environments. For example, finding neutral statistics about Second Life was considerably challenging. Second, information resources such as SL Healthy were found to be rather untrustworthy as many of the listed resources had very little to do with health and many were inactive. Furthermore, there was very little transparency on how the list was constructed. Instead, developers of virtual environments should ensure that reliable, neutral, and up-to-date information is easily available. This would encourage organizations and businesses to invest in creating a presence on such platforms.

For developers of eHealth services, the technical components of virtual worlds such as Second Life enable the building of aesthetically impressive environments that provide rich communication possibilities, which in turn can promote sustained usage and service loyalty [35]. Thus, the lessons learned from Second Life can be useful for developing eHealth initiatives that help individuals to take greater responsibility for their health, promote the building of communities, and offer and receive peer support.

At the same time, learning features, such as controlling the avatar and operating in the virtual environment, requires effort and relationship-specific investment; both are likely to prohibit virtual worlds from diffusing to all segments of the population. The information richness facilitated by virtual worlds may be beneficial in some instances, whereas for those interested in obtain basic information about an illness or other medical condition, a standard website is in most cases a more effective and accessible solution. Furthermore, for building communities and interacting with other people, social networking sites such as Facebook are more convenient and already adopted by a significant proportion of the population. To conclude, based on our experiences and reflections from Second Life, we advise organizations developing eHealth initiatives to rigorously evaluate their features and user interface from a user perspective. Obtaining the user perspective can help the developers of eHealth initiatives to determine when the actual value of the service could be increased by implementing the media-rich features of virtual worlds, or whether a leaner media [36] and simpler user interface, such as a mobile app would be more appropriate to meet the needs of users.

## Multimedia Appendix 1

Summary of health and medical-related information resources in Second Life.

## Multimedia Appendix 2

Data collected from the Second Life Groups.
