# Supplementary figures and images for: Promoting Health in Virtual Worlds: Lessons From Second Life
Source: J Med Internet Res. 2014 Oct 13;16(10):e229. doi: 10.2196/jmir.3177 (PMC4210951; doi:10.2196/jmir.3177)

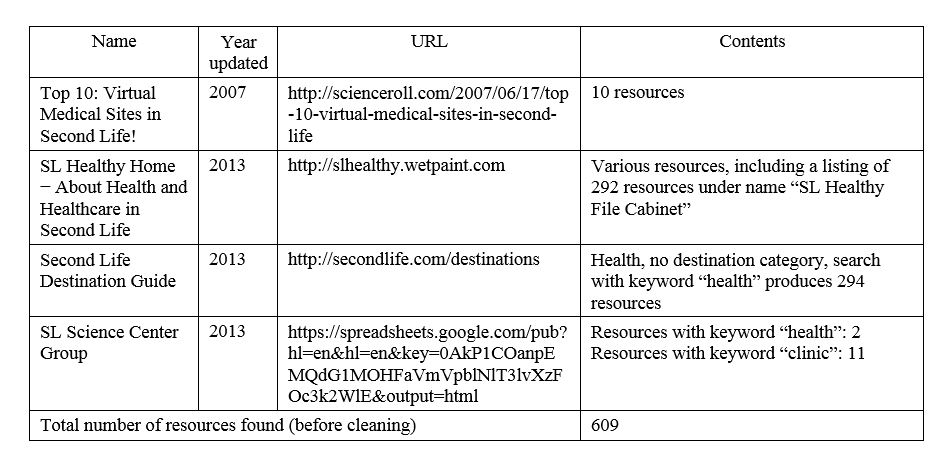

Supplement: Supplementary file 1 [file jmir_v16i10e229_app1.JPG]

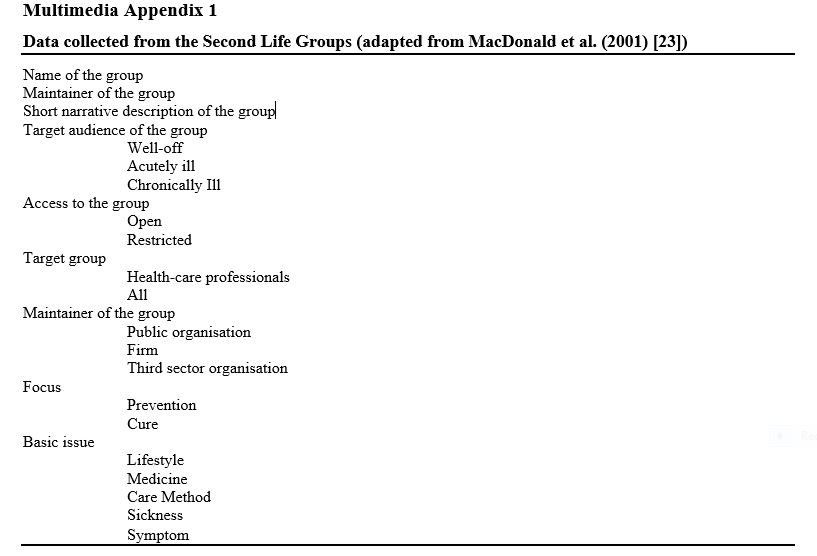

Supplement: Supplementary file 2 [file jmir_v16i10e229_app2.JPG]
